# A Novel Mechanism of S-equol Action in Neurons and Astrocytes: The Possible Involvement of GPR30/GPER1

**DOI:** 10.3390/ijms20205178

**Published:** 2019-10-18

**Authors:** Winda Ariyani, Wataru Miyazaki, Noriyuki Koibuchi

**Affiliations:** 1 Department of Integrative Physiology, Graduate School of Medicine, Gunma University, 3-39-22 Showa-machi Maebashi, Gunma 371-8511, Japan; miya@hirosaki-u.ac.jp; 2 Department of Bioscience and Laboratory Medicine, Graduate School of Health Science, Hirosaki University, 66-1 Hon-cho Aomori, Hirosaki 036-8564, Japan

**Keywords:** soy isoflavone, S-equol, estrogen receptor, GPR30, neuron, astrocyte

## Abstract

S-equol is a major bacterial metabolite of the soy isoflavone daidzein. It is known to be a phytoestrogen that acts by binding to the nuclear estrogen receptors (ERs) that are expressed in various brain regions, including the cerebellum. However, the effects of S-equol on cerebellar development and function have not yet been extensively studied. In this study, the effects of S-equol were evaluated using a mouse primary cerebellar culture, Neuro-2A clonal cells, and an astrocyte-enriched culture. S-equol augmented the dendrite arborization of Purkinje cells induced by triiodothyronine (T_3_) and the neurite growth of Neuro-2A cell differentiation. Such augmentation was suppressed by G15, a selective G-protein coupled ER (GPR30) antagonist, and ICI 182,780, an antagonist for ERs in both cultures. On the other hand, in astrocytes, S-equol induced cell proliferation and cell migration with an increase in the phosphorylated extracellular-signal-regulated kinase 1/2 and F-actin rearrangements. Such effects were suppressed by G15, but not by ICI. These findings indicated that S-equol may enhanced cerebellar development by affecting both neurons and astrocytes through several signaling pathways, including GPR30 and ERs. We here report a novel mechanism of S-equol in cerebellar development that may provide a novel possibility to use S-equol supplementation during development.

## 1. Introduction

S-equol [(S)-3-(4-hydroxyphenyl) chroman-7-ol] is a major metabolite of the soy isoflavone daidzein. It is exclusively produced by intestinal bacteria in response to soy isoflavone intake in some, but not all, humans [1,2]. S-equol has estrogenic activity and binds more tightly to estrogen receptor β (ERβ) than to ERα [2,3]. However, the induction of transcription by S-equol is more potent with ERα than with ERβ in various cell lines [4]. S-equol has also been shown to inhibit the in vivo stimulatory effect of androgen on prostate growth [5]. In Japanese women, S-equol improves the frequency of vasomotor menopausal symptoms, such as night sweats and hot flashes [6]. The natural S-equol supplement SES-5OH stimulates estrogenic transcriptional activity and the proliferation of MCF-7-E10 cells. In addition, neither S-equol nor SES-5OH promotes the progression of implanted breast cancer cells (MCF-7-E10) in ovariectomized mice [7]. Despite the health benefits of S-equol, the underlying molecular mechanism has not yet been fully elucidated. Thus, it is necessary to clarify this mechanism so that it can be employed as an effective pharmaceutical or nutraceutical agent.

S-equol has a slower clearance rate and much higher apparent bioavailability compared to its precursor daidzein [2]. The half-life of S-equol is 7–8 h in healthy adults. While most circulating endogenous estrogen binds to serum proteins such as sex hormone binding globulin, albumin and α-fetoprotein with less than 5% as a free (unbound) form, 49.7% of S-equol circulates in a free form. This ratio is higher compared to its precursor daidzein (18.7% free) [2]. Such reduced protein binding in serum may enhance the availability for receptor binding.

ERs play important roles in the development and functional maintenance of the central nervous system (CNS) [8,9,10]. In the cerebellum, ERs and 17β-estradiol (E2) are involved in Purkinje cell dendrite growth, spines, and synapse formation [10]. E2 also regulates neuronal/glial cell functional maturation, intracellular metabolism, and migration [11]. On the other hand, S-equol may also affect brain development and functional maintenance [12,13,14,15]. It has been shown to protect against focal cerebral ischemia in rats, by upregulating the phosphorylation of Src and gp91(phox) expression levels [12], and reduce oxidase activity and superoxide levels in the brain [13]. S-equol also inhibits microglia-derived clonal cell (BV-2) activation by lipopolysaccharides, increases neurite outgrowth in neuron-derived clonal cells (Neuro-2A), and activates the production of neurotrophins such as nerve growth factor (NGF) in astrocyte-derived clonal cells (C6) [14]. It also prevents synaptic loss by HIV-1 Tat and cocaine mediated synaptopathy in the midbrain and cortical neurons [15]. As discussed above, because S-equol binds to ERs to activate the transcription of target genes, these results indicated that S-equol might affect cerebellar development through ERs.

In addition to nuclear ERs, the study of the orphan G-protein coupled receptor (GPR30, or G-protein coupled ER1, GPER1) is a new research field studying the nongenomic estrogen signaling pathway [16]. Upon binding to estrogen, GPR30 activates several intracellular signal transduction pathways, such as the epidermal-growth-factor-receptor- (EGFR-) mediated pathway to activate extracellular-signal-regulated kinase 1/2 (ERK1/2)- and/or Akt-mediated pathways [17,18,19]. GPR30 is highly expressed in the CNS, including the cerebellum [20]. In the cerebellum, GPR30 is expressed in the Purkinje cell layer, molecular layer, and internal granule cell layer [20]. GPR30 knockout mice are less sensitive to estrogen treatment; these mice show the alterations in anxiety levels and stress responses [21]. GPR30 may also be involved in neuroprotective actions in cerebral ischemia through the activation of PI3-kinase or Src protein kinase [22]. In addition, S-equol inhibited nitric oxide production in lipopolysaccharide-stimulated rat astrocyte cultures, and this effect was partially suppressed by inhibiting GPR30, although the phosphorylation of ERK1/2 by S-equol was not observed [23]. Based on these results, we hypothesized that S-equol may affect cerebellar development through neuronal or astrocytic GPR30, in addition to through ERs.

In order to clarify the mechanism of S-equol action on cerebellar development, we first evaluated its effect on neurite outgrowth, cell proliferation, and cell migration using cultured cerebellar cells and Neuro-2A cells, which are a neuron-derived clonal cell line. We found that S-equol enhanced the dendrite arborization of Purkinje cells in a dose-dependent manner. Such an effect was suppressed by G15, a GPR30 inhibitor, and ICI 182,780 (ICI), an ER inhibitor. S-equol also augmented neurite outgrowth in differentiating Neuro-2A cells with increased levels of ER and GPR30 mRNAs. It also induced the proliferation and migration of cerebellar astrocytes. Unlike neuronal cells, G15, but not ICI, suppressed the effect of S-equol in astrocytes. These results indicated that S-equol may affect cerebellar development through several different pathways.

## 2. Results

### 2.1. S-equol Augmented the Dendrite Arborization of Purkinje Cells in the Primary Cerebellar Culture

**Figure 1:**
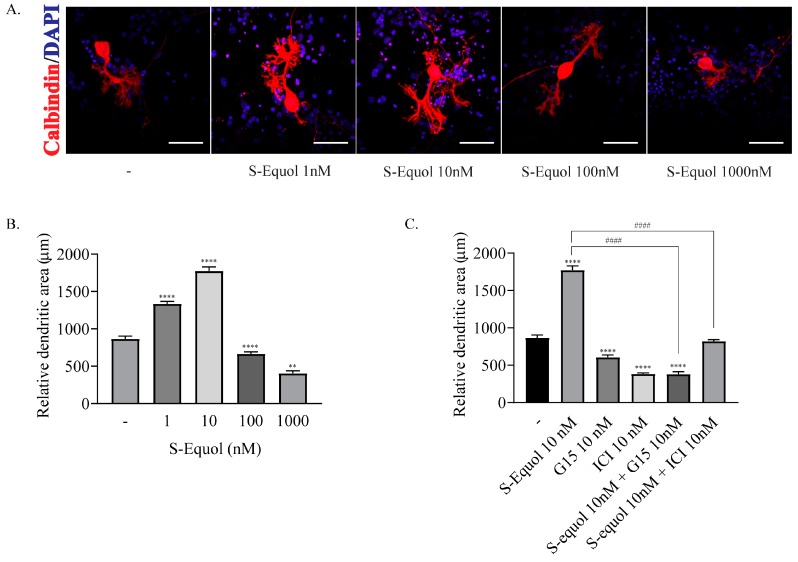
Effects of S-equol on a primary cerebellar culture of Purkinje cells. Cerebellar cells were cultured for 17 days, followed by an immunohistochemical analysis with calbindin (red) and DAPI (4’,6-Diamidino-2-phenylindole) (blue). (**A**) Representative photomicrographs showing the effects of S-equol on the morphology of Purkinje cells. (**B**) Changes in the dendritic areas of Purkinje cells following S-equol (1–1000 nM) treatment. (**C**) Changes in the dendritic areas of Purkinje cells following S-equol, G15 and/or ICI treatment. Dendritic areas were quantified using ImageJ software (NIH). The bars indicate 50 μm. Data are expressed as the mean ± SEM (*n* = 15 determinations) and are representative of at least three independent experiments. ****p < 0.0001, and **p < 0.005, indicate statistical significance according to Bonferroni’s test compared with control (-). ^####^*p* < 0.0001, indicates statistical significance according to Bonferroni’s test compared with S-equol.

We examined the effect of S-equol in the primary cerebellar culture. Dendrite arborization of Purkinje cells was induced by adding 10^−9^ M triiodothyronine (T_3_) to the culture medium. S-equol was added at various concentrations to the culture medium and cells were cultured for 17 days. Then, the cells were fixed and immunocytochemistry of calbindin was performed to visualize Purkinje cells. S-equol augmented the dendrite arborization of Purkinje cells at concentrations of 1 nM and 10 nM, whereas higher concentrations of S-equol did not have such an effect (Figure 1A,B). Next, we examined the effect of S-equol with GPR30 or ER antagonist. Exposure to 10 nM of G15, a GPR30 antagonist, or ICI, an ER antagonist, suppressed S-equol-augmented dendrite arborization of Purkinje cells (Figure 1C). These results indicated that S-equol augmented the dendrite arborization of Purkinje cells through both ERs and GPR30 pathways.

### 2.2. S-equol Augmented Neurite Outgrowth in Neuro-2A Cells

**Figure 2:**
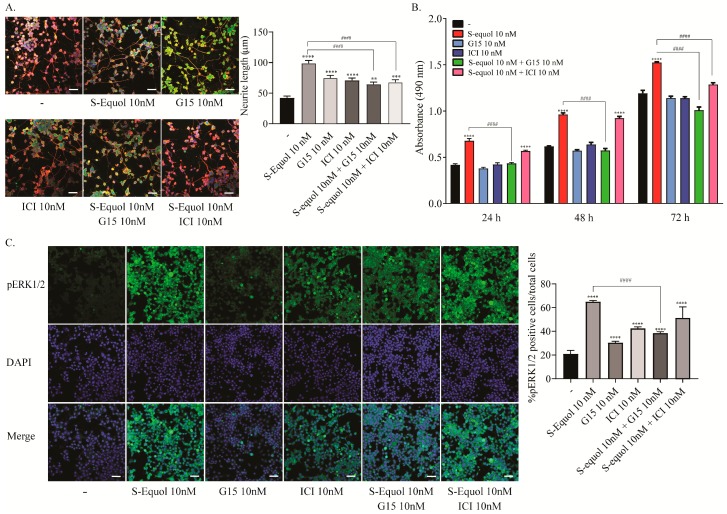
Effects of S-equol on the differentiation, proliferation and phosphorylation of ERK1/2 of Neuro-2A cells. Neuro-2A cells were induced to differentiate by serum starvation and culturing for one to three days, followed by an immunohistochemical analysis with doublecortin (green), β-tubulin III (red), and DAPI (blue). (**A**) Representative photomicrographs showing the effects of S-equol, G15, and/or ICI on the differentiation of Neuro-2A cells. The right panel shows the changes in neurite lengths after exposure. The neurite lengths were quantified using ImageJ Fiji software (NIH). The bars indicate 50 μm. (**B**) Time-dependent changes in the effects of S-equol, G15, and/or ICI on cellular proliferation. Neuro-2A cells were exposed to S-equol, G15, and/or ICI, for 24, 48, and 96 h, respectively. Cell viability was determined with an MTS assay and the number of viable cells was calculated as a percentage of the control viability. (**C**) Representative photomicrographs showing the immunohistochemistry for pERK1/2 and DAPI staining to examine the effect of S-equol, G15, and/or ICI on pERK1/2 expression. The right panel shows the changes in the percentages of pERK1/2 positive cells after exposure. The bars indicate 50 μm. Data are expressed as the mean ± SEM and are representative of at least three independent experiments. ****p < 0.0001, ***p < 0.001, and **p < 0.005, indicate statistical significance according to Bonferroni’s test compared with control (-). ^####^*p* < 0.0001, indicates statistical significance according to Bonferroni’s test compared with S-equol.

In cerebellar primary cultures, thyroid hormone is essential for inducing Purkinje cell dendritogenesis. Thus, whether the effect of S-equol is mediated by ER or TR cannot be clarified. Furthermore, because primary cultures contain several different subsets of cells, the action may be induced by the interaction of such cells. In order to examine the mechanism of action of S-equol during development, we used a Neuro-2A cell differentiation model. To induce differentiation, the concentration of fetal bovine serum (FBS) (TH-depleted) in the culture medium was reduced to 1%. Such serum deprivation induced Neuro-2A differentiation, as shown by the β-tubulin III expression (Supplementary Figure S1). S-equol (10 nM) enhanced neurite outgrowth two fold compared to the controls. Both ICI and G15 (10 nM) treatment suppressed S-equol-augmented neurite growth during Neuro-2A differentiation (Figure 2A). These results are almost the same as those found in Purkinje cells. Using this cell type, we further confirmed the expression of GPR30 and ER at protein and mRNA levels (Supplementary Figure S2). Furthermore, we performed a 3-(4,5-dimethylthiazol-2-yl)-5-(3-carboxymethoxyphenyl)-2-(4-sulfophenyl)-2*H*-tetrazolium (MTS) cell proliferation assay and immunocytochemistry for phosphorylated ERK1/2 (pERK1/2), which is a downstream protein in the GPR30 signalling pathway, to confirm the activation of GPR30 pathway. Low doses of S-equol (10 nM) increased cell proliferation of Neuro-2A cells at 24, 48, and 72 h after exposure. These effects were suppressed by G15. On the other hand, ICI suppressed S-equol-induced proliferation after only 72 h of exposure (Figure 2B). The number of pERK1/2 positive cells was also increased by S-equol, and the S-equol-induced phosphorylation was suppressed by G15 (Figure 2C). These results indicated that S-equol induces neurite extension in Neuro-2A cells through both ER and GPR30, whereas the proliferation may be mainly induced through GPR30.

### 2.3. S-equol Increased the Proliferation of Astrocytes

**Figure 3:**
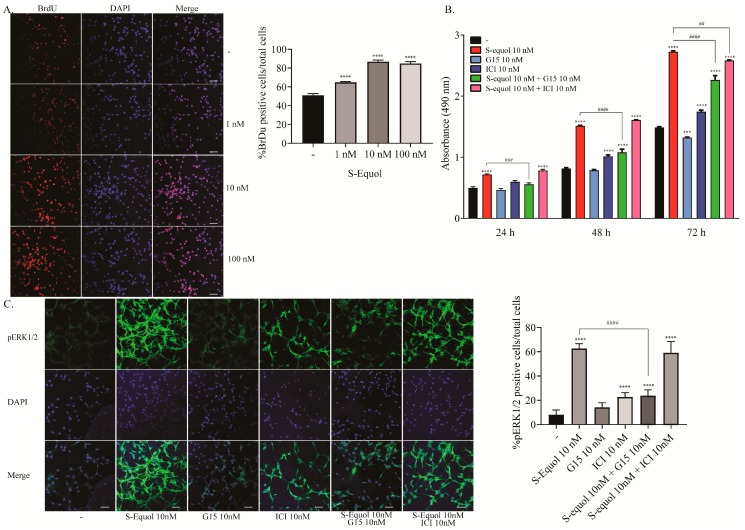
Effects of S-equol on mouse cerebellar astrocyte proliferation. Mouse primary cerebellar astrocytes were cultured for seven days followed by a BrdU incorporation assay, MTS cell proliferation assay, and immunohistochemical analysis with pERK1/2 and DAPI staining. (**A**) Representative photomicrographs showing the effects of S-equol on the BrdU incorporation assay. The right panel shows the changes in the percentages of BrdU positive cells after exposure. (**B**) Time-dependent changes in the effect of S-equol, G15, and/or ICI on cellular proliferation. Astrocytes were exposed to S-equol, G15, and/or ICI for 24, 48, and 96 h, respectively. Cell viability was determined with an MTS assay and the number of viable cells was calculated as a percentage of the control viability. (**C**) Representative photomicrographs showing the immunohistochemistry for pERK1/2 and DAPI staining to examine the effect of S-equol, G15, and/or ICI on pERK1/2 expression. The right panel shows the changes in the percentages of pERK1/2 positive cells after exposure. The bars indicate 50 μm. Data are expressed as the mean ± SEM (*n* = 50 determinations) and are representative of at least three independent experiments. ****p < 0.0001, indicates statistical significance according to Bonferroni’s test compared with control (-). ^####^*p* < 0.0001, ^###^*p* < 0.001, and ^##^*p* < 0.005 indicate statistical significance according to Bonferroni’s test compared with S-equol.

In order to examine the effect of S-equol on astrocyte proliferation, we performed a BrdU incorporation assay, MTS cell proliferation assay, and immunocytochemistry for pERK1/2. S-equol increased the proliferation in a dose-dependent manner (1–100 nM) as showed by a BrdU incorporation assay (Figure 3A). Next, we performed an MTS cell proliferation assay to examine time-dependent changes. We also used GPR30 and ER inhibitors to examine which pathway is responsible for the S-equol action. S-equol increased the cell proliferation of astrocytes at 24, 48, and 72 h after exposure. G15 (10 nM) suppressed S-equol-induced proliferation. However, ICI (10 nM) weakly suppressed the proliferation only after 72 h of exposure, (Figure 3B). The number of pERK1/2 positive cells increased at 30 min after S-equol exposure, and these effects were suppressed by G15 (Figure 3C). These results demonstrated that S-equol induced astrocyte cell proliferation mainly through the GPR30 signaling pathway.

### 2.4. S-equol Increased the Invasion, Lamellipodia Formation, and Rearrangement of Cortical F-actin Activity in Astrocytes

**Figure 4:**
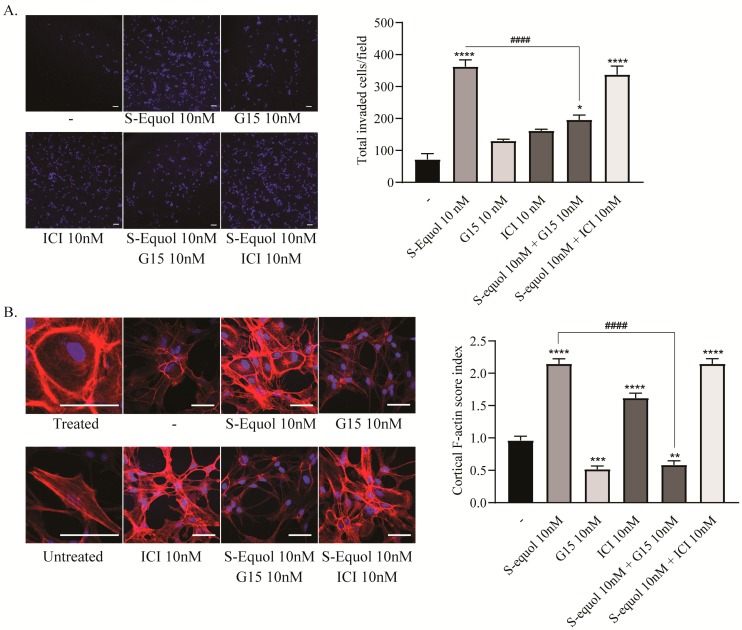
Effects of S-equol on mouse cerebellar astrocyte invasion and F-actin rearrangement. Mouse primary cerebellar astrocytes were cultured for seven days, followed by invasion assays and immunohistochemical analysis with phalloidin F-actin (red) and DAPI (blue). (**A**) Representative photomicrographs showing the invaded cells stained by DAPI (blue) to examine the effects of S-equol. The right panel shows the changes in the total number of invaded cells after 16 h of exposure. The total number of invading cells was quantified using ImageJ software (NIH). The bars indicate 50 μm. (**B**) Representative photomicrographs showing the immunohistochemistry for F-actin and DAPI staining to examine the effects of S-equol on actin polymerization. Astrocytes were exposed to S-equol, G15, and/or ICI for 30 min after serum-starved for 6 h. The right panel shows the changes in CFS index. The bars indicate 50 μm. Data are expressed as the mean ± SEM and are representative of at least three independent experiments. ****p < 0.0001, ***p < 0.001, **p < 0.005, and *p < 0.05, indicate statistical significance according to Bonferroni’s test compared with control (-). ^####^*p* < 0.0001, indicates statistical significance according to Bonferroni’s test compared with S-equol.

In order to further examine the mechanism of S-equol-induced astrocyte migration, we also performed invasion assay and immunocytochemistry for F-actin. As shown in Figure 4A, 10 nM of S-equol increased the invasion of cerebellar astrocytes and the invasion was suppressed by 10 nM of G15. The observed increase in the migration and invasion of cells lead to further examine of the F-actin rearrangement as an indicator of focal adhesions. We performed immunocytochemistry for F-actin and measured the cortical F-actin score (CFS) index. S-equol (10 nM) increased stress fiber formation and CFS index in cerebellar astrocytes. Furthermore, the effects were suppressed by 10 nM of G15, but not by ICI (Figure 4B). These results indicated that GPR30 may play an important role in the migration of astrocytes induced by S-equol.

## 3. Discussion

In our study, we examined the effects of S-equol in the development of neuron and astrocyte. We found that S-equol augmented dendritogenesis, neuritogenesis, proliferation, and migration. While S-equol-induced dendritogenesis and neuritogenesis were suppressed by both G15 and ICI, proliferation and migration were suppressed only by G15. These results suggested the involvement of different signal transduction pathways in mediating the S-equol action. Our results showed the novel action of S-equol in promoting neural/glial development.

It is well known that S-equol exhibits an estrogenic activity. It has been suggested that its actions are mainly exerted by binding to nuclear receptors, mainly ERα and ERβ, which promote estrogen-mediated transcription in many cell lines [3,4]. Binding of ligands to ERs leads to the shuttling of the ligand–ER complex to the nucleus and induces the transcription of target genes through the classical genomic pathway [24,25]. Although the mechanism of action of S-equol to induce transcription through ER has not been clarified, we may speculate that S-equol may direct interactions with ERs through their ligand binding domain, because the action was inhibited by ICI, a complete ER antagonist that inhibits estrogen-mediated transcription and nucleocytoplasmic shuttling of ERs [25,26]. However, the action of S-equol may also be exerted through another pathway, because G15, which is a GPR30 antagonist, also inhibited its action in the proliferation and migration of astrocytes.

Recently, several lines of evidence have shown that estrogen also acts on the receptors, which are located at extranuclear sites, including the plasma membrane, endoplasmic reticulum, Golgi apparatus, and dendritic spines of neurons [27,28,29,30]. Among such pathways, GPR30, activates multiple intracellular signaling pathways that regulate various cellular functions [27,28,29,30]. The ligand-activated GPR30 stimulates the EGFR-mediated signal transduction pathway to activate ERK1/2- and/or Akt-mediated pathways [17,18,19]. A previous study showed the possibility that S-equol may interact with GPR30 in astrocytes and inhibit the production of lipopolysaccharide-induced nitric oxide [23]. In that study, however, although G15 suppressed the effect of S-equol, the effect was partial and phosphorylation of ERK1/2 was not induced by S-equol. Therefore, the involvement of S-equol in astrocytes function was not fully elucidated. On the other hand, in these present studies, G15, a GPR30 inhibitor, significantly suppressed the Purkinje cell dendritogenesis, Neuro-2A neurite growth, and the proliferation and migration of astrocytes. The expression of GPR30 in neurons, including cerebellar Purkinje cells, has been reported in a previous study [20]. Protein and mRNA expression levels were confirmed in cerebellar astrocytes and Neuro-2A cells (Supplementary Figure S2). We also confirmed the increased phosphorylation of ERK1/2, an intracellular protein located downstream of GPR30-mediated signaling. Furthermore, although there has been no report confirming the direct binding of S-equol to GPR30, genistein, which also belongs to isoflavone family can bind to GPR30 with approximately 15% potent affinity compared to estradiol [31]. These results are consistent with our hypothesis that S-equol may bind to GPR30 and activate its signaling pathways to induce functional and morphological alteration of neurons and astrocytes.

Thyroid hormone-activated Purkinje cell dendritogenesis and differentiation-activated Neuro-2A neuritogenesis were augmented by S-equol. Not only ICI but also G15 inhibited such augmentation, indicating the possible involvement of both nuclear ER- and GPR30-mediated pathways. The mechanism of cross-talk between nuclear ER and GPR30 has not fully clarified [32]. They may act in a parallel manner to regulate certain cellular functions, such as intracellular calcium mobilization [33], independently through an independent signaling pathway such as c-fos expression in ovarian cancer cells [34], or in the same signaling pathway in an ordered manner, such as NF-κB-mediated transcription in human monocytes [35]. On the other hand, although the inhibition of either pathway significantly inhibited the S-equol action in neurons, the mode of cross-talk between the two receptors cannot be clarified. Furthermore, such cross-talk was not seen in astrocytes, although both receptors appeared to be expressed. The cross-talk of signalling may depend on the specific cellular function or context.

The cerebellum is known to be a target for estrogen [10]. The effect of estrogen during cerebellar development may be mainly mediated by local estradiol synthesis [36]. Estrogen regulates Purkinje cell dendrite arborization, spines and synapse formation and parallel fiber-Purkinje cell neurotransmission [10,36,37,38,39,40]. In addition, the involvement of membrane-associated receptor ERα and ERβ, for estradiol in the neurite and axon growth is also considered [41]. Binding of estradiol to this receptor activated the MAP kinase pathways, that requires TrkB receptor and leads to an increase in the NGF and brain-derived nerve growth factor (BDNF) levels [41]. In our study, we showed that S-equol may affect Purkinje cell morphogenesis and cerebellum-derived astrocyte proliferation and migration through a genomic and/or nongenomic pathway. It should be noted that, these effects were observed in range of concentration of 1–10 nM. This is within the range of physiological concentration that can be achieved by normal intake of soy-contained food [42]. In addition, S-equol with concentration higher than 100 nM (10–100 times) showed suppression in dendrite arborization of Purkinje cells. This suppression may be caused by toxic effect of S-equol. Previous studies have reported that S-equol treatment with concentrations higher than 2 orders above the physiological range showed inhibit effect in cell proliferation of cancer cell lines [42,43], indicating too much intake of S-equol may cause adverse effect in cellular function. Whether the effect seen in in the present study is profitable or adverse to health has not been determined. However, considering various reports showing the health-promoting effect of a polyphenol-rich diet [6,13,44,45], S-equol may be a potential effective supplement to promote brain development in fetuses and newborns. Further study is necessary to clarify such a possibility.

## 4. Materials and Methods

### 4.1. Chemicals

S-equol and G-15 were purchased from Cayman Chemical (Ann Arbor, MI, USA). ICI 182,780 was purchased from Sigma (St. Louis, MO, USA). The purity of all chemicals was above 98%.

### 4.2. Primary Cerebellar Culture

The animal experimentation protocol in the present study was approved by the Animal Care and Experimentation Committee, Gunma University (19-024, 17 December 2018), and all efforts were made to minimize animal suffering and the number of animals used.

Pregnant mice were purchased from Japan SLC (Hamamatsu, Japan) and newborn mice were euthanized under isoflurane anesthesia at birth. Details of the culture methods have been described previously [46,47]. Briefly, cerebellar tissue was digested with 0.2 units/mL papain (Worthington, Lakewood, NJ, USA) in PBS containing 0.2 mg/mL l-cysteine, 0.2 mg/mL bovine serum albumin (Intergen Company, Purchase, NY, USA), 5 mg/mL glucose, and 0.02 mg/mL DNase I (Sigma, St. Louis, MO, USA) for 25 min with continued shaking at 36.5 °C. Dissociated cells were suspended in a serum-free medium and then plated at a density of 3 × 10^5^ cells/0.3 mL in the wells of chamber slides (Lab-Tek 8 mm diameter wells; Nunc International, Rochester, NY, USA) that were precoated with 0.1 mg/mL poly-l-lysine (Sigma). Then, at 16–24 h after plating, S-equol was added to the culture medium containing T_3_ (10^−9^ M). One-half of the medium was replaced with fresh medium every three days. The mixed cerebellar cells were cultured in a 5% CO_2_ incubator at 37 °C for 17 days. The effect of dimethyl sulfoxide was excluded using control and experimental media at a final concentration of 0.01% and by avoiding freezing and thawing.

Cells were rinsed three times with PBS, fixed with 4% PFA, and blocked with 2% FBS [46]. Cells were incubated with mouse monoclonal anti-calbindin-28K antibody (1:200; Sigma), donkey anti-mouse IgG (H + L) secondary antibody, Alexa Fluor^®^ 594 conjugate (1:200; Thermo Fisher Scientific, Inc, Waltham, MA, USA). They were then inspected under a laser confocal scanning microscope (Zeiss LSM 880; Carl Zeiss Microscopy GmbH, Jena, Germany). The cell nuclei were also stained with DAPI. The total area covered by the dendritic tree on 20–50 randomly selected Purkinje cells per experiment was determined to quantify dendrite arborization by tracing the outline of the cell and its dendritic branches before computing the total area using ImageJ software (NIH, Bethesda, MD, USA). Data are presented as the mean ± standard error of the mean (SEM). A typical result from one experiment is shown graphically. More than three independent experiments were performed. The results were consistent for each experiment.

### 4.3. Mouse Neuro-2A Culture and Induction of Differentiation

Neuro-2A cells, which are mouse neuroblastoma-derived clonal cells, were maintained in Dulbecco’s modified Eagle’s medium (DMEM) supplemented with 10% FBS and antibiotics (100 U/mL penicillin, and 100 µg/mL streptomycin) with 5% CO_2_ at 37 °C. The serum was stripped of hormones by constantly mixing with 5% (*w*/*v*) AGXI-8 resin (Bio-Rad, Hercules, CA, USA) and powdered charcoal prior to ultrafiltration [48]. Neuro-2A cells were seeded at a density of 1 × 10^5^ cells/1 mL per well the day before the experiment in DMEM + 10% FBS on 6- or 24- well plates coated with poly-l-lysine. Differentiation was induced by serum deprivation as described previously [49,50]. In short, the DMEM +10% FBS culture medium was changed to a DMEM +1% FBS culture medium to induce differentiation. On the next day, the culture medium was changed to prewarmed DMEM containing 1% FBS with or without the indicated concentration of S-equol, 10 nM of G15 and/or 10 nM of ICI to trigger differentiation, and it was then cultured for one to three days. The cultures were then harvested for RT-qPCR and immunocytochemistry. The cells were rinsed three times with PBS, fixed with 4% PFA, and then blocked with 2% FBS. The cells were then incubated with mouse monoclonal anti-β-tubulin III (neuronal) antibody and rabbit anti-doblecortin (C-terminal) antibody (1:200; Sigma) followed by donkey anti-mouse IgG (H + L) secondary antibody, Alexa Fluor^®^ 594 and donkey anti-rabbit IgG (H + L) secondary antibody, Alexa Fluor^®^ 488 conjugate (1:200; Thermo Fisher Scientific, Inc.). Cell nuclei were also stained with DAPI. The cells were then inspected under a laser confocal scanning microscope (Zeiss LSM 880, Carl Zeiss Microscopy GmbH). Neurite length measurements were performed using ImageJ Fiji (NIH).

### 4.4. Primary Culture of Cerebellar Astrocyte

A primary culture of mouse cerebellar astrocytes was prepared as previously described [51,52] with slight modifications. Briefly, P1 mouse cerebella were dissected and digested with 2.5% trypsin (Wako, Osaka, Japan) in HBBS (Wako) for 30 min with continued shaking at 37 °C. The cells were resuspended in an astrocyte culture medium (DMEM high-glucose, 10% heat-inactivated FBS, 1% penicillin/streptomycin), and 10–15 million cells were plated on 10 cm dishes coated with Collagen-I (Iwaki, Tokyo, Japan). The cells were incubated at 37 °C in the CO_2_ incubator. On DIV3, astrocyte culture medium was replaced with PBS. Dishes were then shaken by hand for 0.5–1 min until only the adherent monolayer of astrocytes was left. PBS was then replaced with a fresh astrocyte culture medium. Astrocytes were harvested on DIV7 with 0.25% trypsin 1 mmol/L Na.EDTA (Wako), and then plated on 12- or 24-well dishes. The quality of primary culture of cerebellar astrocyte was examined by immunocytochemistry with astrocyte marker including S100β and GFAP (Supplementary Figure S3). The cells were used for cell proliferation, invasion or F-actin activity assays.

### 4.5. BrdU Incorporation Assay

We performed a BrdU incorporation assay using the method described in the instruction manual of the BrdU staining and BrdU assay protocol from Abcam (Cambridge, UK). Astrocytes were plated at a density of 1 × 10^5^ cells/mL on Poly-L-lysine-coated coverslips in 24-well plates and then incubeted with serum-starved DMEM for 6 h. The cells were treated with or without S-equol (1–100 nM) together with 10 µM BrdU labeling solution (Abcam) for 24 h. The cells were then washed with PBS and fixed with 4% PFA. The DNA was hydrolyzed using 2 M HCl for 1 h at room temperature and then blocked with 2% FBS. The cells were incubated with rat anti-BrdU antibody [BU1/75 (ICR1)] (ab6326) (1:200; Abcam) and a donkey anti-rat IgG (H + L) secondary antibody, Alexa Fluor^®^ 594 conjugate (1:200; Thermo Fisher Scientific, Inc.). Cell nuclei were also stained with DAPI. The cells were then inspected under a laser confocal scanning microscope (Zeiss LSM 880, Carl Zeiss Microscopy GmbH).

### 4.6. Cell Proliferation Assay

We performed an MTS cell proliferation assay, as described in the instruction manual of the Promega Technical Bulletin CellTiter 96^®^ AQueous One Solution Cell Proliferation Assay. The conversion of MTS into aqueous soluble formazan was caused by the succinic dehydrogenase found in the metabolically active mitochondria. DIV7 of cerebellar astrocytes was harvested and plated at a density of 1 × 10^5^ cells/0.1 mL on 96-well plates and then incubated in the presence of S-equol, ICI and/or G15 for 24, 48, and 72 h, respectively. After exposure to these compounds, the MTS reagent was added to the medium. Absorbance, represents the mitochondrial metabolic activity, was measured at 490 nm using a microplate reader (Bio-Rad). Relative values were calculated as percentages of the values obtained from the normal control group. All MTS studies were repeated at least three times in triplicate. Data are presented as the mean ± SEM of one representative experiment that was performed in triplicate.

### 4.7. Matrigel Invasion Assay

The in vitro invasion assay was performed using a 24-well Millicel^®^ hanging cell culture insert and a Corning^®^ Matrigel^®^ matrix according to the manufacturer’s instructions. In brief, 1 × 10^5^ astrocytes/mL were seeded in free-serum DMEM in the upper chamber. The lower chamber was filled with DMEM and S-equol, ICI, and/or G15. After 16–18 h of incubation, noninvading cells in the upper chamber were removed with a sterile cotton swab. The filters of inserts were fixed with 4% PFA and stained with DAPI. The number of invading cells on the lower surface of the filter was counted.

### 4.8. Lamellipodial Formation and CSF Index

Astrocytes were cultured on poly-L-lysine-coated coverslips and serum-starved DMEM for 24 h. The cells were then treated with either S-equol, ICI or G15 for 30 min. The cells were then washed with PBS and fixed with 4% PFA. They were then blocked with 2% FBS. Cells were incubated with CytoPainter Phalloidin-iFluor 594 reagent and the cell nuclei were also stained with DAPI. The cells were then inspected under a laser confocal scanning microscope (Zeiss LSM 880, Carl Zeiss Microscopy GmbH). The degree of cytoskeletal rearrangement was examined using the CFS index [53]. The CFS index was determined on the basis of at least three independent experiments. Briefly, the F-actin cytoskeletal reorganization for each cell was scored on a scale ranging from 0 to 3, which was based on the degree of cortical F- actin ring formations (0, no cortical F-actin, normal stress fibers; 1, cortical F-actin deposits below half the cell border; 2, cortical F-actin deposits exceeding half the cell border; 3, complete cortical ring formatting and/or total absence of central stress fiber. A minimum of 50 cells were examined from each group in each independent experiment, and the CFS index for S-equol, G15, and/or ICI treated astrocytes was the average score of the counted cells ± SEM.

### 4.9. Statistical Analysis

All statistical analyses were performed with GraphPad Prism 8 (https://www.graphpad.com/), and the data are expressed as the mean ± SEM of three individual experiments performed in triplicate and analyzed using an analysis of variance (ANOVA). Post-hoc comparisons were made using Bonferroni’s test. A *p*-value of <0.05 was considered to be significant.

## 5. Conclusions

**Figure 5:**
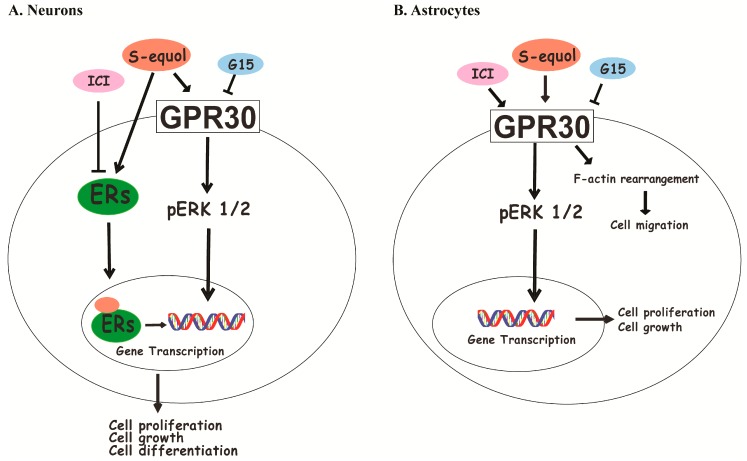
The proposed mechanism of S-equol in cerebellar development through both genomic and nongenomic actions. S-equol enters the cells and may bind (↓) to the cytoplasmic, nuclear ERs or GPR30 to induce gene transcription, which leads to cell growth and augmentation of dendrite arborization in neurons (**A**). These effects were suppressed by an ER inhibitor, (i.e., ICI (┴)). On the other hand, S-equol also activated the GPR30 (↓) signaling pathway through the activation of phosphorylation of ERK1/2, enhanced cell proliferation, and induced F-actin rearrangement to increase cell migration in astrocytes (**B**). These effects were suppressed by the GPR30 inhibitor G15 (┴).

In summary, S-equol, which is a major metabolite of daidzein, augmented dendritogenesis of cerebellar Purkinje cells, neuritogenesis of Neuro-2A cells, and proliferation and migration of cerebellar astrocytes. These effects were suppressed by inhibiting GPR30 or nuclear ER functions, indicating that S-equol may affect cerebellar development through genomic and/or nongenomic pathway. Our study has provided the possibility that S-equol may potentially be an effective supplement to promote brain development (Figure 5).

## Supplementary Materials

Supplementary Materials can be found at https://www.mdpi.com/1422-0067/20/20/5178/s1.
