# Supplementary material for: A Novel Mechanism of S-equol Action in Neurons and Astrocytes: The Possible Involvement of GPR30/GPER1
Source: Int J Mol Sci. 2019 Oct 18;20(20):5178. doi: 10.3390/ijms20205178 (PMC6829462; doi:10.3390/ijms20205178)
Supplement: Supplementary file 1 [file ijms-20-05178-s001.zip › Supplementary_materials/Supplementary Materials Figure Legends.docx]

**Supplementary Materials Figure Legends**

**Supp. Figure S1.** Serum deprivation induced Neuro-2A differentiation**.** (A) Representative photomicrographs showing the effects of serum deprivation on the differentiation of Neuro-2A cells. Neuro-2A cells induced differentiation by serum starvation and then culturing for one to three days. After fixation, the cells were incubated with mouse monoclonal anti-β-tubulin III (neuronal) antibody and rabbit anti-doblecortin (C-terminal) antibody, (1:200; Sigma), followed by incubation with donkey anti-mouse IgG (H+L) secondary antibody, Alexa Fluor® 594 and donkey anti-rabbit IgG (H+L) secondary antibody, Alexa Fluor® 488 conjugate (1:200; Thermo Fisher Scientific, Inc). The cell nuclei were also stained with DAPI. The cells were then inspected under a laser confocal scanning microscope (Zeiss LSM 880, Carl Zeiss Microscopy GmbH, Jena, Germany).

**Supp. Figure S2.** Protein and mRNA expression levels in the cerebellar astrocytes and Neuro-2A cells**.** (A) Representative photomicrographs showing GPR30 expression in astrocytes and Neuro-2A cells. Cells were immunostained with polyclonal anti-GPER(GPR30) antibody produced in rabbit (1:200; Alemone Labs, Jerusalem, Israel) and DAPI. (B) Change in ER mRNA expression levels in astrocytes and Neuro-2A cells due to exposure to S-equol. The total RNA was isolated using QIAzol® Lysis Reagent (QIAGEN, US), and the RNA was then reverse-transcribed using ReverTra Ace® qPCR RT Master Mix (TOYOBO Bio-Technology, Japan) according to the instructions in the manual provided by the manufacturer. RT-PCR was performed using THUNDERBIRD® SYBR® qPCR Mix (TOYOBO Bio-Technology) as described in the instruction manual, and a StepOne RT-PCR System (Applied Biosystems). The RT-PCR protocol for all genes involved denaturation for 20 s at 95°C, followed by amplification for 3 s at 95°C and for 30 s at 60°C (40 cycles). All experiments were repeated three times, using independent RNA preparations to confirm consistency. The mRNA levels were normalized by the mRNA level of GAPDH. Data are expressed as the mean ± SEM and are representative of at least three independent experiments. *^*^p* < 0.05, ^****^*p* < 0.0001, indicates statistical significance according to Bonferroni’s test compared with controls. (C) List of qRT-PCR primer sequences.

**Supp. Figure S3.** Representative photomicrographs showing immunocytochemistry for S100β or GFAP in cultured cerebellar astrocytes (DIV9). Astrocytes were cultured on Poly-L-lysine-coated cover slip in 24-well plates. After fixation, cells were incubated with rabbit polyclonal anti-S100β antibody or rabbit polyclonal anti-GFAP antibody, (1:200; Frontier Institute co., ltd. Hokkaido, Japan), followed by incubation with donkey anti-rabbit IgG (H+L) secondary antibody, Alexa Fluor® 594 (1:200; Thermo Fisher Scientific, Inc). The cell nuclei were stained with DAPI. The cells were then inspected under a laser confocal scanning microscope (Zeiss LSM 880, Carl Zeiss Microscopy GmbH, Jena, Germany).
